# Polysensitive radiation recall dermatitis following Prevnar 20 vaccination

**DOI:** 10.1016/j.jdcr.2024.08.004

**Published:** 2024-08-30

**Authors:** Evadne Rodriguez, Benjamin W. Casterline, James Roller

**Affiliations:** a School of Medicine, University of Missouri - Columbia, Columbia, Missouri; b Department of Dermatology, University of Missouri - Columbia, Columbia, Missouri

**Keywords:** breast cancer, chemoradiotherapy, dermatitis, immunization, medical oncology, Prevnar 20, Prevnar 20 vaccination, radiation oncology, radiation recall, radiation recall dermatitis, radiodermatitis, vaccination

## Introduction

Radiation recall dermatitis (RRD) is a poorly characterized phenomenon defined by an acute inflammatory reaction triggered by the administration of a systemic agent arising focally in areas of previously irradiated skin and soft tissue.^1^ First reported in association with actinomycin D, most cases arise after administration of chemotherapeutic agents including doxorubicin, docetaxel and paclitaxel, gemcitabine, and capecitabine.^1^^,^^2^ RRD developing after treatment with immunomodulators, aromatase-inhibitors, antibiotics, statins, and COVID-19 vaccinations has also been described.^3^, ^4^, ^5^, ^6^ The exact incidence of RRD is unknown, however it is estimated to occur in approximately 6% of individuals who receive radiation therapy.^3^ The clinical features of RRD vary from mild eruptions exhibiting dry desquamation and pruritus, to more severe instances of swelling, maculopapular lesions, ulceration, and skin necrosis.^1^^,^^3^ We describe a novel case of RRD after Prevnar 20 vaccination in a patient who had previously experienced RRD during adjuvant chemotherapy.

## Case report

**Fig 1:**
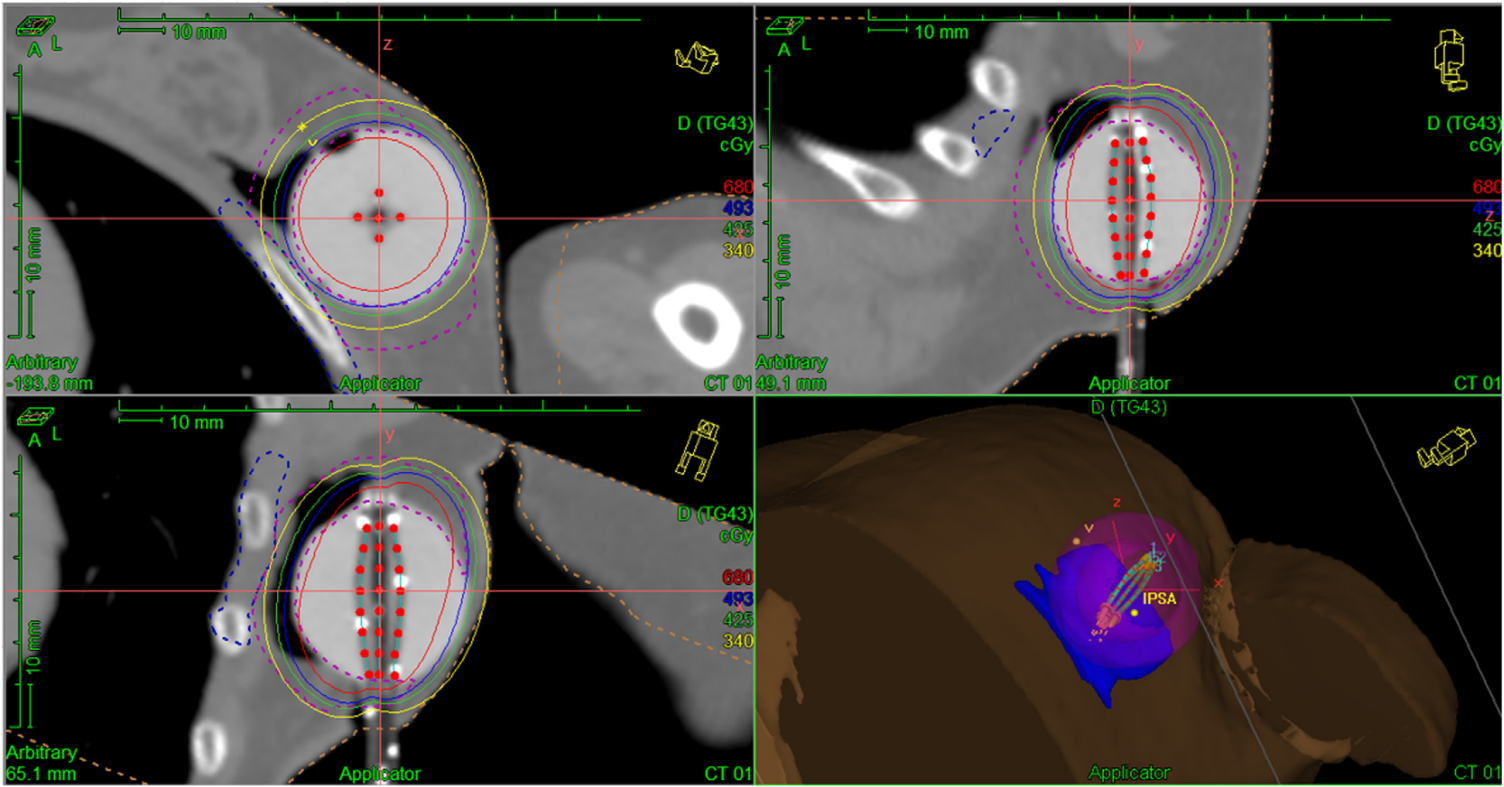
Radiation field.

We report the case of a 74-year-old woman who was diagnosed with stage IA mucinous carcinoma of the left breast and treated with lumpectomy, adjuvant accelerated partial breast irradiation (34 Gy in 3.4 Gy fractions) delivered with the MammoSite device (Fig 1), followed 1 week later by adjuvant chemotherapy with paclitaxel and trastuzumab. The patient was not evaluated for BRCA mutation. One daughter was subsequently found to be negative for cancer-associated BRCA mutations. There is no family history of any collagen or connective tissue disorders.

During the second week of chemotherapy, RRD developed, manifesting as pruritic, indurated erythema of the left breast and lateral chest. This was followed by desquamation and resolved after 5 weeks. The patient completed 12 weeks of paclitaxel and trastuzumab, then continued trastuzumab to complete 52 weeks of therapy. She ultimately completed 5 years of adjuvant letrozole and is without tumor recurrence.

**Fig 2:**
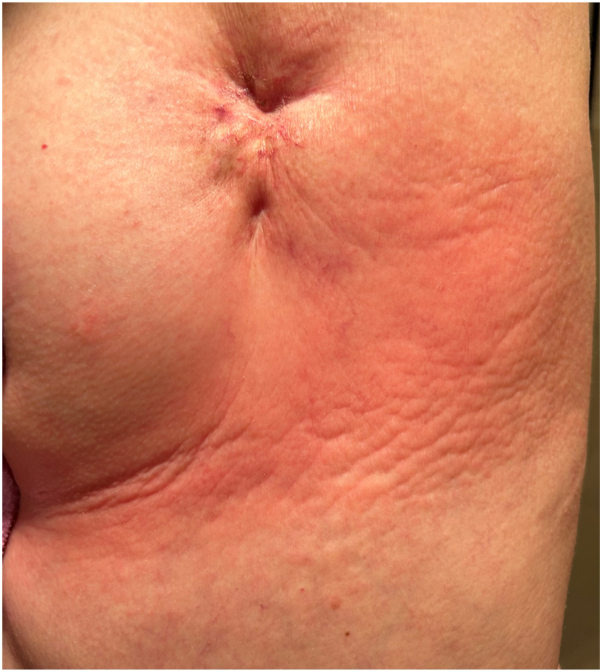
Left chest radiation recall dermatitis at scarred surgical pocket and brachytherapy port.

**Fig 3:**
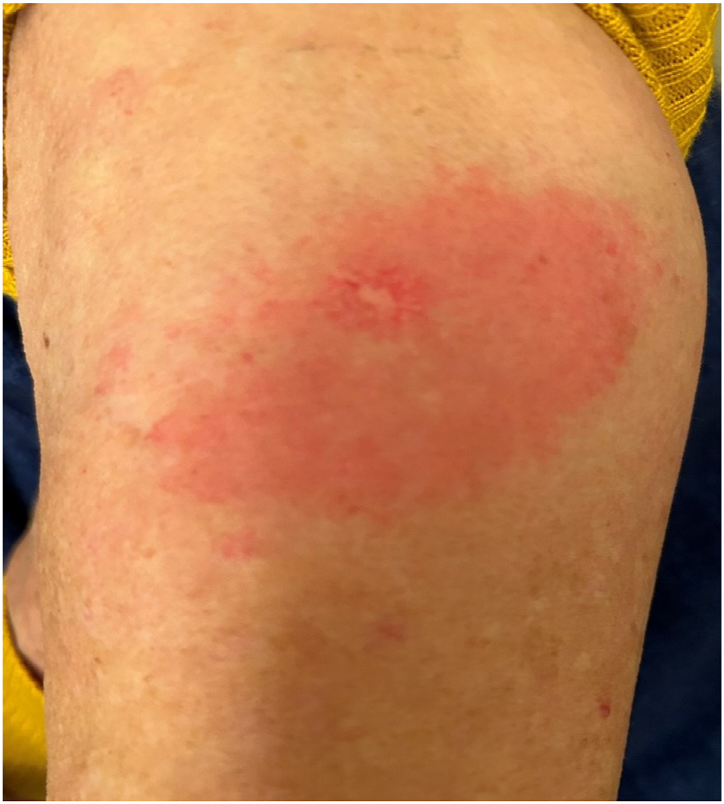
Vaccination site reaction.

Seven years after her initial diagnosis of breast cancer, the patient received a Prevnar 20 vaccination. Within 6 hours of the immunization, pruritic erythema developed over the left chest. The pruritus worsened over the next 2 days with deepening erythema and induration. She was diagnosed with RRD (Fig 2) and treated with clobetasol ointment and menthol gel with clearance of the eruption after 7 days. Six days from the onset of the eruption over her chest, an indurated, pruritic, erythematous patch developed at her vaccination site (Fig 3). It was similarly treated with clobetasol ointment and menthol gel and cleared within 1 week. At the time of this episode of radiation recall, her medications included spironolactone, carvedilol, atorvastatin, sacubitril/valsartan, calcium and vitamin D supplementation, and a multivitamin.

The patient’s vaccination history was significant for a Prevnar 13 vaccination without cutaneous reaction 1 year before her diagnosis of breast cancer. Additionally, she received a flu, respiratory syncytial virus, and fourth COVID-19 vaccination 2 months before the Prevnar 20 vaccination without side effects.

## Discussion

In this report we document a case of RRD to multiple different agents, an unusual phenomenon known as polysensitivity. Alternative diagnoses including carcinoma erysipeloides, erysipelas, and contact dermatitis were considered. The patient’s clinical history, timing of the eruption after vaccination, limited distribution to prior site of radiation, and definitive response to steroids support the diagnosis of RRD. Although the patient in this case developed RRD to paclitaxel and trastuzumab therapy and Prevnar 20, the patient had no adverse reaction to Prevnar 13, respiratory syncytial virus, influenza, or COVID-19 vaccinations. This implies a specific cross-reactivity between either paclitaxel or trastuzumab and one or multiple capsular polysaccharides present in Prevnar 20.

There are no definitive characteristics of the agents that have been shown to induce radiation recall, insight into which patients may be affected, and by which agents they may or may not be affected.^1^ Soyfer et al^4^ reported the first case series of 2 patients who presented with RRD after messenger RNA COVID-19 vaccination. Since their report a handful of additional case reports have been published featuring RRD after vaccination to COVID-19 spike protein using messenger RNA, inactivated virus and adenovirus vector technologies.^5^^,^^6^ According to Sepaskhah et al,^5^ there had been no reported events of RRD associated with any vaccinations before the onset of COVID-19 vaccinations.

Radiation therapy causes cutaneous immune compromise through the generation of free radicals, irreparable breaks in cell DNA, and by triggering an inflammatory cascade.^3^ Therefore radiation therapy may upregulate tissue-resident inflammatory cells and cytokines, such as CD8^+^ T cells, interferon gamma, and tumor necrosis factor-alfa, which are then amplified by exposure to a triggering agent, such as chemotherapeutic drugs. A similar mechanism has been proposed for fixed drug eruption (FDE).^1^^,^^3^^,^^7^ In FDE, interleukin 15 dependent CD8^+^ memory T cells reside in the basal layer of resting FDE lesions and are either activated into a CD56^+^ natural killer-like phenotype or recruit circulating CD56^+^ lymphocytes that migrate to the epidermis where they release granzyme B and perforin to induce epidermal necrosis after exposure to a specific drug.^8^ The self-limited nature of the reaction is explained by sequestration of interleukin 2 due to infiltration of CD25^+^ regulatory T cells.^8^ FDE has been reported in response to vaccination and shows polysensitivity.^9^^,^^10^

Prior hypotheses to explain how previously irradiated tissue may become susceptible to RRD include vascular damage, increased sensitivity of epithelial stem cells, epithelial stem cell depletion, and FDE-like sensitization.^10^ These proposals may account for certain features of RRD, including normal appearance and function of the skin before the triggering agent, agent-specific responses, speed of onset, occasional improvement of cutaneous reactions with agent rechallenge, and occurrence even with noncytotoxic drugs.^10^ As in FDE, however, a mechanism by which intramuscular vaccination could induce RRD at distant sites has not been described. In this case, the patient’s prior immunization against capsular polysaccharides from the 13 *Streptococcus pneumoniae* serotypes shared between Prevnar 13 and Prevnar 20 suggests that extant humoral immunity against these antigens may underlie the presentation via antibody-dependent natural killer cell activation or another antibody-dependent pathway.

## Conflicts of interest

None disclosed.
